# Exploring the long-term impact of a nurse-midwife mentorship intervention in Neno district, Malawi: a secondary data analysis of maternal and neonatal complications

**DOI:** 10.1186/s44263-024-00107-9

**Published:** 2024-11-11

**Authors:** Miranda Rouse, Isaac Mphande, Nelson Mwale, Sitalire Kapira, Mphatso Sayenda, Mc Geofrey Mvula, Maria Openshaw, Esnath Kapito, Martha Kutsamba, Daniel Maweu, Ashley Mitchell, Madhavi Dandu, Anna Muller, Alden Hooper Blair, Kimberly Baltzell

**Affiliations:** 1 University of California, San Francisco, Institute for Global Health Sciences, Mission Hall, 550 16Th Street, Third Floor, Box 1224, San Francisco, CA 94158 USA; 2 Partners In Health/Abwenzi Pa Za Umoyo, P.O. Box 56, Neno, Malawi; 3 GAIA Global Health, P.O. Box 51428, Limbe, Malawi; 4 Partners In Health, 25 Saquee Drive, Off Wilkinson Road, Freetown, Sierra Leone; 5 MGH Institute of Health Professions, School of Nursing, 36 1St Avenue, Boston, MA 02129 USA; 6 Kamuzu University of Health Sciences, Private Bag 360, Chichiri, Blantyre 3 Malawi; 7 Neno District Health Office, Ministry of Health, P.O. Box 52, Neno, Malawi; 8 University of California Global Health Institute, 1111 Franklin Street, Oakland, CA 94607 USA; 9 Seattle Children’s Hospital, 4800 Sand Point Way NE, Seattle, WA 98105 USA; 10 University of California, San Francisco, School of Nursing, N431Y, 2 Koret Way, Box 0606, San Francisco, CA 94143 USA

**Keywords:** Obstetric complications, Neonatal complications, Data reporting, Nursing, Midwifery, Sub-Saharan Africa

## Abstract

**Background:**

There is critical need to strengthen the global nursing and midwifery workforce. This is especially true in Malawi where they are the primary providers of obstetric and neonatal care. In Neno district, Malawi, in 2017, we implemented an intensive training and longitudinal bedside mentorship intervention for nurses and midwives. From inception, there was a pre-planned project target completion after 5 years, including a staged handover to local ownership after 3 years. The objective of this study was to assess differences in reported maternal and neonatal complications following project completion and handover to local leadership.

**Methods:**

The project was a partnership between an academic institution and local nongovernmental organization. In October 2020, the intervention was handed over and maintained by the local organization with limited support from the academic institution. Data from January 2019 to May 2023 were extracted from the Malawi District Health Information Software 2. Bivariate analyses explored differences in the pre- and post-handover periods for all government-collected, birth-related variables. The “pre-handover” period encompassed January 2019 to September 2020 and “post-handover” from October 2020 to May 2023. Multivariate linear regression explored outcomes while controlling for health facility.

**Results:**

Data were collected from 10 health facilities in the district and included a total of 23,259 births. Overall, there were few significant changes between periods. Exceptions were in reporting of antepartum hemorrhage (*p* < 0.01), maternal sepsis (*p* = 0.01), and manual removal of the placenta (*p* < 0.01), where we observed decreases in reporting. There was a significant decrease in the reporting of neonatal sepsis (*p* = 0.01) in the bivariate analysis, which remained only borderline significant in the multivariate model (*p* = 0.05). Where differences occurred, they were associated with improvements in reported identification of complications and provision of associated emergency care.

**Conclusions:**

Few differences in reported maternal and neonatal complications between the periods suggest positive impact of the intervention was sustained following project handover and transition of activities to local leadership. These findings strengthen support for longitudinal mentorship as a pivotal component for skill retention after training. Transparent partnerships which include pre-determined end points and time for handover of activities to local ownership are crucial components for sustainability.

## Background

Despite nurses and midwives making up the largest cadre of health providers around the globe, they are still in critical demand [1]. There is a global nursing shortage of over five million, a global midwifery shortage of over 900,000, and the UNFPA’s State of the World’s Midwifery Report 2021 found sub-Saharan Africa has the most severe deficit [2–4]. This workforce shortage is caused by a myriad of challenges including poor working conditions, lack of physical resources, inadequate pay, limited support at the bedside, few opportunities for professional development, and constraints imposed on governments by international funders [5–7]. In turn, these challenges contribute to high attrition rates for the profession, worse quality of care, and high maternal and neonatal mortality rates [8].

The Republic of Malawi, a landlocked country in southern Africa, ranks among countries with the highest rates of maternal and neonatal mortality [9, 10]. In 2020, Malawi recorded a maternal mortality ratio of 381 per 100,000 live births, and neonatal mortality has remained around 27 per 1000 live births for many years [11, 12]. While the country has worked to improve outcomes and increase the number of well-trained providers, policies by the International Monetary Fund (IMF) often interfere with the hiring of new nurses and midwives [5]. There are also persistent human resource gaps across all cadres, districts, and health care levels within Malawi’s public sector [13]. The Ministry of Health has a 51% vacancy rate of which registered nurse midwives (RNM) constitute 40% and nurse midwife technicians (NMT) 54% of the vacancy rate [13]. This, in turn, leaves existing providers to face extreme staffing shortages, further hindering their ability to grow professionally and support new or less experienced providers. In addition, nurses and midwives are the primary providers of obstetric and neonatal care and may be the first and only provider a patient encounters across the pregnancy care continuum [1, 14]. Thus, strengthening the nursing and midwifery workforce must be a priority if there is any hope to achieve Sustainable Development Goal 3.1 to reduce the global maternal mortality ratio to less than 70 per 100,000 live births [15, 16].

One common approach to strengthening the knowledge and skills of clinical providers is training, yet there is a plethora of research that shows training alone is not enough to embed new attitudes, behaviors, or practices toward improving patient outcomes [17, 18]. Evidence has found that short-course training without follow-up support is ineffective and often a waste of valuable resources [18–21]. However, there are data to suggest intensive clinical didactic trainings must be followed by long-term mentorship to reinforce learnings. Emerging evidence has found improved retention of skills and increased clinical competence of providers who participate in a low-dose, high-frequency training model as compared to traditional didactic group learning without follow-up support. Literature on the low-dose, high-frequency model included mobile mentorship by providers via telephone and text message. While in some cases this was found to be a helpful method to continued learning, it cannot replace in-person, bedside mentorship [22–24].

In 2017, we established a partnership between a nongovernmental organization (NGO) working in Malawi and a US-based academic research institution to implement a combined intervention of intensive training with longitudinal bedside mentorship for nurses and midwives. The partnership has expanded to multiple countries, but this study will focus on the longest-established project within the partnership. The intervention was first implemented in Neno district, Malawi, to strengthen the skills of nurses and midwives working in the obstetric and neonatal space. The unique mentorship component of the intervention differs from traditional supervision in that an expert nurse-midwife mentor works both clinically alongside providers at the bedside as well as imparting professional support beyond clinical skills. From the beginning, there was a planned, staged handover of the project to local leadership after 3 years, with a project completion goal of 5 years. Initial research into this intervention found improved data quality and integrity through accurate reporting of obstetric and neonatal complications and there is evidence to support improved skill retention [25–28].

A pivotal component of global health practice is collaboration and partnership, with the goal of sustaining progress. The recent recognition of the longstanding inequity in global health partnerships has resulted in frameworks for equitable and sustainable collaboration [29, 30]. Values highlighted in these frameworks include strong local leadership, mutual trust, and transparency. Indeterminant endpoints and a lack of transparency around funding duration pose challenges for sustainability; therefore, successful global health partnerships should start with a mutually agreed upon end date [31, 32]. This foresight encourages early planning within externally funded projects for long-term sustainability.

Therefore, the objective of this study was to identify differences in reported maternal and neonatal complications following project handover to local leadership. We hypothesized there would not be any significant differences in the reporting of obstetric and neonatal complications given continued longitudinal mentorship to embed and retain skills [33, 34].

## Methods

This was a secondary data analysis to measure the impact of the intervention following project handover to local leadership. The data in this study included routinely collected maternal and neonatal variables from Malawi Ministry of Health maternal and newborn registers, aggregated monthly at each health facility for reporting into the Malawi District Health Information Software 2 (DHIS2) database [35].

### Study setting

**Fig. 1:**
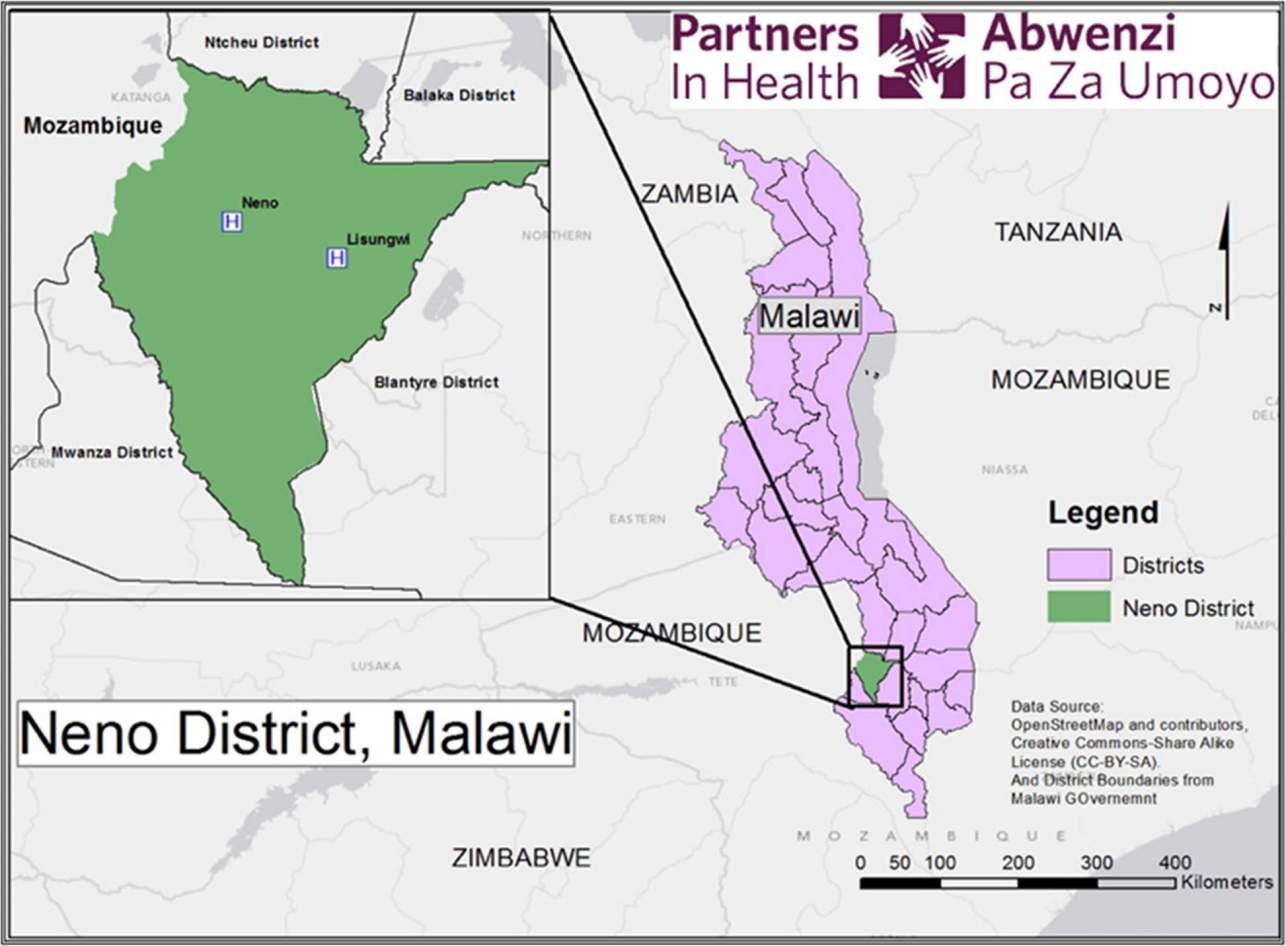
Map of Neno district and health facilities

Neno is a rural district in the Southern Region of Malawi where the local NGO Abwenzi Pa Za Umoyo (APZU), or Partners In Health (PIH) in Malawi, has been working since 2007. In 2017, the short-course training and longitudinal mentorship intervention was implemented in 10 health facilities across the district: eight health centers, one community hospital, and one district hospital (Fig. 1). For purpose of this paper, the term health facility is used to describe the physical entities where individuals seek health care. We use the term heath center to describe the peripheral health facilities which conduct uncomplicated, vaginal deliveries, including breech birth. The term hospital is used to describe the community and district hospitals which have the additional capacity to conduct cesarean birth, vacuum-assisted vaginal birth, and serve as the referral facilities for obstetric and neonatal complications. In addition, the breakdown of providers at these facilities includes RNMs, NMTs, and community midwifery assistants (CMAs). Despite differing levels of education and training for these providers, their skills and experience are not commensurate with degree type. The standard pre-service training requirements for each cadre of provider is as follows: RNM is a 4-year, bachelor-level degree; NMT is a 3-year diploma, and CMA is an 18-month certificate. The health facilities in the district are managed and governed by the Ministry of Health and adhere to national standards.

### Intervention model

In 2017, the US-based academic research institution University of California San Francisco (UCSF) Global Action in Nursing (GAIN) project was invited by APZU and the Neno District Health Office to support improvements in maternal and neonatal outcomes in Neno district. The intervention in this study has been described in detail elsewhere [25–27]. Briefly, it was a nurse-designed intervention to improve nurses and midwives’ clinical practices and quality-of-care through a short-course intensive training followed by longitudinal bedside mentorship. The content of the training was developed by the study team in 2017, prior to the first cohort, and formulated around the World Health Organization Safe Childbirth Checklist. All training materials were adapted for the Malawian setting, aligned with Ministry of Health protocols, and are available open access on the study website [36].

The model uses an asset-based approach to strengthen the skills of in-service providers who are limited in their professional development by structural determinants beyond their control [37, 38]. The novel component of this intervention is the coupling of training with longitudinal bedside mentorship for a minimum of 12 months. Providers from each of the 10 health facilities participated in a 7-to-10-day intensive training which covered topics delivered in the following order: leadership skills, fundamentals of quality improvement, obstetric care delivery, and management of neonatal complications. The duration of the training was adapted from 10 to 7 days after the first cohort per local request to prioritize simulation over didactic learning and reduce the time providers were away from the bedside delivering patient care. Providers who deliver midwifery care at the health facilities were invited to participate in the training. This included those who worked in the antenatal, labor, and postnatal wards. Physicians and clinical officers were not included in this intervention, as they are a different cadre of provider and cannot receive training or mentorship from nurse-midwives per national guidelines. The training was facilitated by a Malawian midwife, a US-based midwife, an American pediatric nurse practitioner who lived and worked in Malawi, and Ministry of Health colleagues, including a local quality improvement expert. Following completion of training, two expert nurse-midwife mentors rotated weekly among the 10 health facilities to provide bedside support and mentorship to providers. In addition, the iterative nature of mentorship allowed for the Malawian team to identify areas to target and bolster mentorship; those initiatives were locally led. Expert nurse-midwife mentors were master’s prepared, experienced providers who were identified and hired initially by the US-based team and then by leadership at APZU. The mentors all held a Master of Science in Nursing and/or Midwifery degree. Bedside mentorship was provided on a weekly basis in which two mentors rotated around the 10 facilities on a biweekly schedule. The number of rotating mentors later increased to four after the third cohort. They would spend approximately 6 h at the facility working side-by-side at the bedside with the providers, giving additional support as needed. A WhatsApp channel was also created so mentors and mentees had a direct line of communication when they were not in-person [39]. The mentorship schedule was established by the leadership to align with activities in the district [31, 40]. Following successful implementation across multiple cohorts, the focus shifted to ensuring the model could be independently sustained by the Malawian team, eventually replacing outside mentors with Neno-based mentors in close collaboration of Ministry-level officials [31]. This period of focus shifting was part of the staged handover plan. By October 2020, the US-based team stepped back from day-to-day activities but continued to support in other areas such as securing funding, providing educational and professional development opportunities for mentors, and analyzing data for research evaluation. The post-handover period allowed the US-based team to support the NGO partner—who is a permanent partner in the district and is guided by the Ministry of Health in all aspects of their work—to explore different funding opportunities. During this period, the partner developed a relationship with a funder directly, thus leading to complete project handover.

### Study terms

Though the intervention was led by expert nurse-midwife mentors and APZU leadership in Neno, the study team in the US provided active support for implementation from 2017 to September 2020. In October 2020, as mentioned above, support continued only in the areas of funding, professional development, and data analysis. In this manuscript, we will refer to the period from January 2019 through September 2020 as “pre-handover” and October 2020 to May 2023 as “post-handover.” The pre-handover period is 22 months, while the post-handover period is 31 months. Additionally, throughout this manuscript, we may use the terms maternal, mother, or woman when referring to birthing persons and their care in deference to the language widely used and accepted in the study setting of Malawi.

### Data collection

**Fig. 2:**
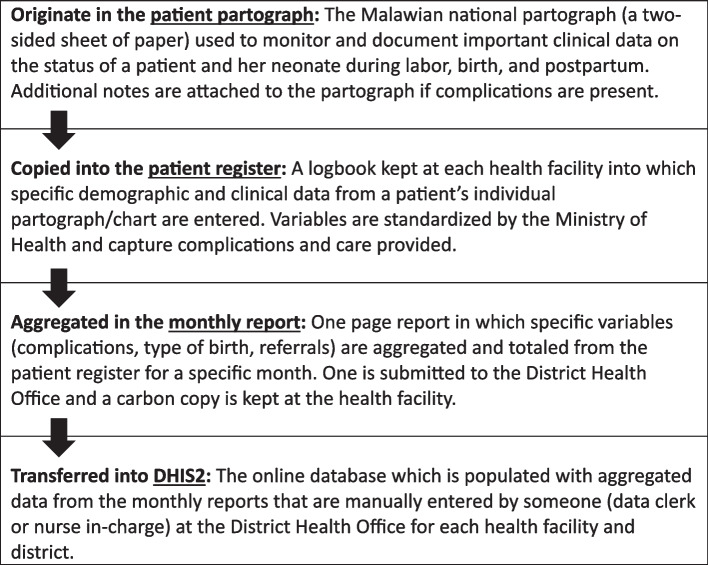
DHIS2 data origin and entry process

The variables of interest for this study came from the Ministry of Health maternal and neonatal registers. Each month, a health facility nurse in-charge aggregates and records the total number for each variable, which is then submitted to the District Health Office in a monthly report and manually entered to DHIS2 (Fig. 2). These variables include maternal complications, associated emergency obstetric care, neonatal complications, associated emergency neonatal care, location and mode of delivery, neonatal survival, and referrals to tertiary care. All maternal and neonatal complications and associated emergency care variables are listed in Tables 2 and 3. Delivery location variables include health facility, in transit, at home and/or with a traditional birth attendant (TBA), or another facility, while mode of delivery include vaginal, breech vaginal, vacuum, or cesarean section. Neonatal survival variables include neonatal death, fresh stillbirth, and macerated stillbirth. Data for this study were extracted in June 2023 from the DHIS2 database. Due to a change in maternal registers at the end of 2018, this study focused on data from 2019 onwards.

Based on prior findings which highlight limited data integrity with the multiple step process, the study team first conducted a rigorous data cleaning process [41]. This included coordination with the expert nurse-midwife mentors to verify missing entries and outliers in the DHIS2 database with the carbon copy of the monthly report kept at each facility. There were five full monthly entries from health facilities, which the team was unable to verify with the carbon copies—these were removed from the dataset.

### Data analysis

Final data were uploaded to the statistical analysis program R version 4.2.1 (2022–06-23) for analyses [42]. Due to the differences in patient volume at study facilities, data from the monthly reports were analyzed as percentages based on the number of births in the facility in a given month. At times, facilities would record complications for a mother who was admitted at the study facility during labor, but who was referred to tertiary care before delivery. The birth itself would then not be counted in the facility’s monthly report. This caused some reported complication percentages to rise slightly above 100%. This has been described elsewhere and in more detail in the discussion section [25, 28].

First, descriptive statistics were used to summarize the distributions of the variables overall and during the pre- and post-handover periods. This showed the data were not normally distributed given the relative rarity of complications and emergency care given. Accordingly, the study team chose a conservative approach to use nonparametric tests and report median and ranges to summarize and show the distribution of variables. Kruskal Wallis and Fisher’s exact tests were conducted to assess for differences between periods with a *p*-value of < 0.05 chosen to denote statistical significance.

Recognizing the potential for provider- and facility-level factors to influence rates of complications, multivariate linear regressions were then conducted to assess the underlying relationship between the handover period and outcomes of interest in the presence of confounders. Potential confounders included individual facility, facility type (health centers versus hospitals), and time; both on their own and as they interact. Due to differences in patient load and average monthly births at health centers versus hospitals, we explored potential differences by facility and facility type (health centers versus hospitals) at both the bi- and multivariate level. Nested models were compared with goodness of fit test, and non-nested models with Bayesian informational criterion (BIC). The final model chosen for this study included only facility, with the district hospital as the reference facility due to highest patient load. As only the two hospitals offered vacuum and cesarean births, a sub-analysis explored the rates of these modes of delivery. The multivariable models are presented with point estimates (pt. est), and 95% confidence intervals (95% CI) to show both strength and directionality of associations.

## Results

Data for this study included 525 monthly reports (209 pre-handover and 316 post-handover) from January 2019 to May 2023 across 10 facilities in Neno district, Malawi. The reports encompassed a total of 23,259 births (9313 pre-handover and 13,946 post-handover). Facilities reported an average of 44.3 births per month, ranging from 3.7 at the least busy facility to 162.1 at the busiest. There were a reported 200 births at the busiest facility in one month and there were 2 months at the least busy facility where no births were reported. Health centers averaged 21.2 births per month, while hospitals reported a mean of 135.6 births per month. The mean number of births per month per facility can be seen in Table 1.

**Table 1:** Summary of health facilities included in this study

Health facility	Mean number of births per month	Median percent of referred cases per month (%)	Configuration of providers
Health center 1	24.38	12.50	1 RNMs, 2 NMTs, 2 CMAs
Health center 2	21.45	14.84	1 RNMs, 1 NMTs, 1 CMAs
Health center 3	3.74	28.57	0 RNMs, 2 NMTs, 2 CMAs
Health center 4	13.00	10.52	1 RNMs, 3 NMTs, 1 CMAs
Health center 5	17.50	13.33	0 RNMs, 2 NMTs, 1 CMAs
Health center 6	40.30	16.66	0 RNMs, 4 NMTs, 0 CMAs
Health center 7	17.0	27.27	0 RNMs, 2 NMTs, 0 CMAs
Health center 8	31.52	3.70	0 RNMs, 1 NMTs, 0 CMAs
Community hospital	109.03	0.88	8 RNMs, 22 NMTs, 0 CMAs
District hospital	162.13	0.00	23 RNMs, 42 NMTs, 0 CMAs

Registered nurses (RNs) have been excluded because they do not have midwifery qualifications and therefore do not provide any obstetric services in antenatal, labor, and postnatal wards

Bivariate and subsequent multivariable findings found minimal to no difference in reported complications and associated care 31 months after project handover, as the majority of findings in this study were not statistically significant. The results from the bivariate and multivariate models are summarized below. As described in the methods, the multivariable model included only facility, which is by the adjusted *p*-value.

There were three monthly reports during the post-handover period with missing variables related to neonatal complications and care. The missing data did not affect the overall distribution.

### Maternal complications and associated emergency obstetric care

Overall, maternal complications were relatively rare at the study facilities, with “none” frequently reported in a given month (Table 2). The most common complication was obstructed/prolonged labor with a median 3.85% prevalence in the pre-handover period and a 3.57% in the post-handover period. Due to low patient volume at some facilities, the prevalence of complications and associated care in a given month could vary dramatically, such as with obstructed/prolonged labor where three cases in 1 month resulted in a 100% prevalence for that month. However, there were two significant decreases in the reported rate of maternal complications between the pre- and post-periods: antepartum hemorrhage (pt. est: − 0.52; 95% CI: − 0.86, − 0.17) and maternal sepsis (pt. est: -0.32; 95%CI: -0.57, -0.06). In the case of associated care, the only significant difference was in manual removal of the placenta (pt. est: − 0.29; 95% CI: − 0.46, − 0.12). All three of these maternal complications and associated care were significant in both bi- and multivariate models controlling for facility, which showed a decrease in their reported occurrence following project handover.

**Table 2:** Median reported percent of maternal complications and care per monthly total births at each facility

	**Pre-handover (total months = 209) Median % [min, max]**	**Post-handover (total months = 316) Median % [min, max]**	**Unadjusted *p*-value**	Adjusted *p*-value*
*Maternal complications*				
Ruptured uterus	0 [0, 7.14]	0 [0, 22.2]	0.932	0.732
Obstructed/prolonged labor	3.85 [0, 100]	3.57 [0, 100]	0.188	0.459
Pre/eclampsia	0 [0, 16.7]	0 [0, 25.0]	0.378	0.361
Antepartum hemorrhage	0 [0, 20.0]	0 [0, 20.0]	0.005	0.004
Fetal distress	0 [0, 16.7]	0 [0, 33.3]	0.643	0.624
None	92.9 [0, 120]	93.5 [0, 133]	0.724	0.762
Maternal sepsis	0 [0, 20.0]	0 [0, 8.33]	0.031	0.015
Retained placenta	0 [0, 15.0]	0 [0, 100]	0.519	0.431
Other	3.26 [0, 100]	3.36 [0, 100]	0.900	0.673
Postpartum hemorrhage	0 [0, 21.4]	0 [0, 50.0]	0.342	0.499
Premature labor	0 [0, 100]	0 [0, 50.0]	0.079	0.630
*Associated emergency obstetric care*				
Anticorticosteroids	0 [0, 100]	0 [0, 50.0]	0.056	0.071
Obstetric antibiotics	0 [0, 40.0]	0 [0, 100]	0.218	0.826
Evacuation of retained products	0 [0, 1.27]	0 [0, 0.990]	0.098	0.061
Anticonvulsives	0 [0, 10.0]	0 [0, 25.0]	0.488	0.766
Blood transfusion	0 [0, 3.57]	0 [0, 2.17]	0.378	0.955
Manual removal of placenta	0 [0, 11.1]	0 [0, 7.69]	< 0.001	0.001
Non-pneumatic antishock garment	0 [0, 5.26]	0 [0, 5.26]	0.158	0.229

^*^Multivariable model accounted for health facility

### Neonatal complications and associated emergency neonatal care

Like maternal complications and care, those reported for neonates were rare overall (Table 3). The most common neonatal complication was birth weight below 2500 g (reported median prevalence pre-handover: 4.76%, post-handover: 3.57%), and the most common emergency care provided was neonatal resuscitation (reported median prevalence pre-handover: 2.38%, post-handover: 1.14%). There was a significant decrease in the reporting of neonatal sepsis (pt. est: − 0.34; 95% CI: − 0.67, − 0.00) in the bivariate analysis, which remained only borderline significant in the multivariate model (pt. est: − 0.33; 95% CI: − 0.67, 0.00). Following adjustment for confounders at the facility level, the multivariate model showed rates of neonatal antibiotics—given in cases of neonatal sepsis among other complications—significantly decreased between the pre- and post-handover periods (pt. est: − 1.21; 95% CI: − 2.21, − 0.20). While not significant at the bivariate level, after adjusting for facility, reported rates of prematurity significantly increased (pt. est: 1.24; 95% CI: 0.05, 2.43) in the post-handover period.

**Table 3:** Median reported percent of neonatal complications and care per monthly total births at each facility

	**Pre-handover (total months = 209)** **Median % [min, max]**	**Post-handover (total months = 316)** **Median % [min, max]**	**Unadjusted *p*-value**	Adjusted *p*-value*
*Neonatal complications*				
Birth asphyxia	3.47 [0, 33.3]	3.14 [0, 50.0]	0.283	0.178
None	86.4 [0, 100]	87.1 [0, 100]	0.594	0.721
Other	0 [0, 16.7]	0 [0, 25.0]	0.591	0.343
Prematurity	2.38 [0, 50.0]	2.68 [0, 66.7]	0.184	0.040
Neonatal sepsis	0 [0, 19.4]	0 [0, 20.0]	0.012	0.050
Weight < 2500 g	4.76 [0, 91.3]	3.57 [0, 62.5]	0.271	0.317
*Associated emergency neonatal care*				
Neonatal antibiotics	0 [0, 79.8]	0 [0, 20.0]	0.074	0.019
Other	0 [0, 100]	0 [0, 10.2]	0.536	0.275
Neonatal resuscitation	2.38 [0, 33.3]	1.14 [0, 50.0]	0.224	0.150

^*^Multivariable model accounted for health facility

### Delivery location and mode

There were no significant differences found in the rates of birth location (as documented in the patient register as either “this facility,” “other facility,” “in transit,” or “at home/TBA”) between the pre- and post-handover periods. Likewise, there were no significant results in the bivariate analysis for modes of delivery. The prevalence of breech birth significantly decreased (median prevalence pre-handover: 1.66%, post-handover: 1.11%; pt. est: − 0.56; 95% CI: − 1.05, − 0.06) in the multivariate model; the change was only associated with one of the health centers. A sub-analysis for cesarean and vacuum birth at hospitals—the only facilities qualified to offer such procedures—found a significant increase (median prevalence pre-handover: 17.3%, post-handover: 21.6%; pt. est: 4.14; 95% CI: 2.22, 6.06) in cesarean deliveries following project handover. There were no significant differences for vacuum birth.

### Neonatal survival and referrals

There were no statistically significant differences in neonatal survival. The mean rate of neonatal death pre-handover was 0.79% and it decreased to 0.31% post-handover. The median rate in both periods was 0%. The mean reported rate of fresh stillbirth was 0.39% in the pre-handover period, and it decreased to 0.28% in the post-handover period. Macerated stillbirth had a mean reported rate of 0.31% pre-handover, and it increased to 0.72% post-handover. All neonatal survival variables (neonatal death, fresh stillbirth, macerated stillbirth) had a median reported rate of 0% in both the pre- and post-handover periods. There were also no significant differences between the pre- and post-handover periods for referrals from health centers to hospitals (median reported referrals from health centers to hospitals: 15.7% pre-handover, 12.5% post-handover; *p* = 0.18).

## Discussion

The primary objective of this study was to examine differences in the identification and treatment of key complications and care following project handover of a training and mentorship intervention [25, 27, 28]. We were specifically interested in what, if anything, changed following project handover of all activities from the academic partner to local, NGO leadership. The study findings suggest the reporting of obstetric and neonatal complications and delivery of emergency care remained stable after the complete handover.

While this study focused on the reporting of complications and emergency care, it is important to note the uptake of longitudinal bedside mentorship by local partners, which was sustained for the entire duration of the project and the post-handover period. It continues to be maintained as standard practice in the district. As mentioned previously in the methods section, complete handover was achieved when the NGO partner was connected to a funder directly to sustain project activities in partnership with the Ministry of Health. This study found no significant difference in the identification of critical complications between the periods. Reporting of complications remained high, and without change in negative outcomes or referrals to higher-level facilities, suggesting potential timely identification and care provision.

In the few instances where statistically significant change was noted, the directionality was towards continued improvements in care. This corresponded to ongoing trainings and quality improvement projects initiated by clinical leaders at APZU, such as Basic Emergency Obstetric and Neonatal Care (BEmONC) courses for nurses and midwives [43]. The significant decrease in the reporting of neonatal sepsis corresponded with a significant decrease in in the provision of neonatal antibiotics. It is possible this concomitant change is related to a reduction in neonatal infection due to training and mentorship, hence the reduction in the reporting of neonatal sepsis cases and concurrent antibiotic prescribing. This plausible explanation would strengthen evidence for the impact longitudinal mentorship had on providers’ ability to detect complications early and treat them in a timely fashion or potentially prevent them altogether [28, 44–46].

During analysis of mode of delivery, we found a significant increase in cesarean births between the pre- and post-handover periods. Given this change, we expected to see a subsequent increase in referrals, yet there were no significant changes in referrals between the project periods. The change in cesarean births could be due to a two-month period in 2019 which cesarean sections were not conducted at the community hospital because the surgical theater was under construction and an anesthetist was on sick leave. While there was a decrease in breech vaginal birth, significance was driven by one health facility which had the lowest average number of births per month. There was only one breech birth reported in all 53 months included in this study, and it was during a month when only four births occurred at the facility, thus accounting for 25% of births. Furthermore, although the reporting of prematurity significantly increased in the multivariate model, there was no significant change in the reporting of neonatal death, suggesting better care of premature neonates [47]. The median rate of prematurity during the post-handover period was 2.68% of births, which is well below the Malawian national average [48]. Further exploration is needed to understand this difference. It is of note that there was an increase in space and staff for the neonatal intensive care unit at the district hospital between 2019 and 2021 which could support the improvement in neonatal survival [47]. As seen in Table 3 in the results, there was a higher median report rate of neonatal asphyxia compared to the median reported rate of neonatal resuscitation. We would expect to see the rate of complication—neonatal asphyxia—to track with associated care—neonatal resuscitation. Despite a slight difference in median reported rate, the range of both do match. Further investigation into the discrepancy is warranted.

While there is no agreed upon distinction between mentorship versus coaching and supportive supervision, all have been found to be a strong predictor in the prevention of skill and knowledge regression after training and have even been found to improve job satisfaction and workforce retention [18, 19, 33]. Clinical mentorship for nurses and midwives has also been shown to increase provider confidence, reinforce complication identification and management, and improve overall service delivery [46, 49]. In-service mentorship is especially important in Malawi due to the unique workforce shortage challenges. In Malawi, the public health sector is limited in their ability to hire available providers, leaving recent nurse-midwife graduates to experience a prolonged period of unemployment before deployment by the Ministry of Health [50]. This period of time out of clinical practice strengthens the argument for in-service mentorship. In addition, pre-service clinical training is limited by the lack of human resources in the health facilities and faculty, often causing student nurses to act as part of the workforce during school [51]. Our prior work and the results from this study suggest that clinical improvements made through longitudinal mentorship are sustainable across the transition of project leadership. The mentorship rotation continued after the intervention beyond the twelve-month period and is maintained as standard practice in the district currently.

There are a few limitations to note. First, the multiple step process data entry into DHIS2, highlighted in Fig. 2, may lead to reduced data integrity. Prior research by the study team found there is high potential for data loss into DHIS2 given the onerous reporting system [25, 41]. With this as evidence, we made sure to conduct a rigorous data cleaning strategy to mitigate as many errors as possible. While this was a time-consuming process, it did help to rectify multiple outliers in the dataset through review of the hard copy monthly reports. Given this strategy and previous studies which have evidence of improved reporting and data integrity from the intervention, we believe the dataset was reflective of the district’s rate of complications and care during the time period [28]. Also, since the transition after 2018 to allow for the reporting of multiple complications, the reporting of none was found to be recorded in addition to other complications, thus making the total percentage of births with no complications reported rise above 100 percent. The constant transfer of providers between districts by Ministry officials to fill vacancies may have impacted the number of providers who completed the intensive training portion of the intervention. However, regardless of their training status, all providers across study facilities received bedside mentorship to refine their clinical skills. While there was not a separate control group for this study, handover of project leadership was one of a few unique factors during the pre- and post-handover periods. Previous study found that there was no significant decrease in births occurring at health facilities during COVID-19 pandemic [26]. This strengthens the case for mentorship and collaborative partnerships as key factors of sustained progress.

With an increasing focus on equitable partnerships in global health, stakeholders, including funders, are prioritizing investment in efforts that demonstrate long-term impact through capacity building, both for clinical providers and mentorship of researchers in low- and middle-income countries [29, 52]. Sustainability requires a shift towards activities that result in measurable change and the meaningful involvement of local organizations in interventions from the beginning [31, 40]. Training alone is not enough to change provider practices. Instead, programs requiring longer-term investment, including the one described in this study which combines training with mentorship, are necessary to embed the confidence and skills needed to improve care quality and ultimately patient outcomes [40, 53, 54]. Two key aspects of project sustainability included (1) initial establishment of partnership from the start and (2) the intention to have a staged handover of all project activities within 3 to 5 years. Implementation protocols and training materials for the intervention in this study are available open access on the study website (https://gainproject.ucsf.edu/files/operations-manual) [36]. It is an adaptable program which can be replicated in other low-resource settings following the establishment of strong partnership with the local governing body for health services. This intervention has been successfully implemented in one other district in Malawi and two other countries in sub-Saharan Africa.

Unfortunately, traditional funding models do not allow for analysis after a meaningful amount of time following project completion or handover. This study is unique in evaluating the sustainability of a training and longitudinal mentorship intervention on maternal and neonatal complications and care following handover to local leadership. We found the reporting of complications remained primarily unchanged in the 31-month period after project handover to local partners. The lack of significant difference in the pre- and post-handover periods indicates practices gained from the intervention were sustained and strengthens support for longitudinal mentorship as a critical component for improving clinical practices and, eventually, patient outcomes.

## Conclusions

The results of this study provide strong evidence for the sustained impact of training and mentorship interventions on maternal and neonatal complication identification and care when collaboratively implemented in low-resource settings like rural Malawi. Transparent partnerships, which include a determined end point, time for handover of activities, and local leadership and ownership of projects, are also crucial components of sustainability [55].

Training followed by long-term, hands-on mentorship is now standard practice in this study setting, having expanded to clinical areas beyond maternal and neonatal health [56]. Given the pivotal role nurses and midwives have in providing obstetric and neonatal care, especially in low-resource settings, efforts to improve maternal and neonatal health must be centered around not only improving skills but continuing to support skill development and ability to provide timely and responsive care.

## Data Availability

The data utilized in this study are not publicly available and the researchers were not given permission to share the data as it belongs to the government of the Republic of Malawi. Data access requests must be made directly to the Malawi Ministry of Health via the website: dhis2.health.gov.mw. The code on which the conclusions of the paper rely has been made available via Github at this link: https://zenodo.org/records/14020079 [57].

## Abbreviations

RNM: Registered nurse midwife
NMT: Nurse midwife technician
NGO: Nongovernmental organization
DHIS2: District Health Information Software 2
PIH: Partners In Health
APZU: Abwenzi Pa Za Umoyo
UCSF: University of California San Francisco
GAIN: Global Action in Nursing
CMA: Community midwifery assistant
TBA: Traditional birth attendant
IRB: Institutional Review Board
BIC: Bayesian informational criterion
BEmONC: Basic Emergency Obstetric and Neonatal Care
